# Biosynthesis and assessment of antibacterial and antioxidant activities of silver nanoparticles utilizing *Cassia occidentalis* L. seed

**DOI:** 10.1038/s41598-024-57823-3

**Published:** 2024-03-27

**Authors:** Arvind Arya, Pankaj Kumar Tyagi, Sachin Bhatnagar, Rakesh Kumar Bachheti, Archana Bachheti, Mansour Ghorbanpour

**Affiliations:** 1 Department of Chemistry, Graphic Era University, Dehradun, India; 2 Noida Institute of Engineering and Technology, Greater Noida, India; 3 Nanotechnology Centre of Excellence, Addis Ababa Science and Technology University, P.O. Box 16417, Addis Ababa, Ethiopia; 4 Department of Environmental Science, Graphic Era University, Dehradun, India; 5 Department of Medicinal Plants, Faculty of Agriculture and Natural Resources, Arak University, Arak, 38156-8-8349 Iran; 6 Institute of Nanoscience and Nanotechnology, Arak University, Arak, 38156-8-8349 Iran

**Keywords:** Silver nanoparticles, TEM, SEM, XRD, EDX, Antioxidant potential, Antibacterial potential, Pathogenic bacteria, Chemical biology, Plant sciences, Chemistry

## Abstract

This research explores the eco-friendly synthesis of silver nanoparticles (AgNPs) using *Cassia occidentalis *L. seed extract. Various analytical techniques, including UV–visible spectroscopy, transmission electron microscopy (TEM), scanning electron microscopy (SEM), X-ray diffraction (XRD), and energy dispersive X-ray spectroscopy (EDX), were employed for comprehensive characterization. The UV–visible spectra revealed a distinct peak at 425 nm, while the seed extract exhibited peaks at 220 and 248 nm, indicating the presence of polyphenols and phytochemicals. High-resolution TEM unveiled spherical and oval-shaped AgNPs with diameters ranging from 6.44 to 28.50 nm. The SEM exhibiting a spherical shape and a polydisperse nature, thus providing insights into the morphology of the AgNPs. EDX analysis confirmed the presence of silver atoms at 10.01% in the sample. XRD results unequivocally confirm the crystalline nature of the AgNPs suspension, thereby providing valuable insights into their structural characteristics and purity. The antioxidant properties of AgNPs, *C. occidentalis* seed extract, and butylated hydroxytoluene (BHT) were assessed, revealing IC_50_ values of 345, 500, and 434 μg/mL, respectively. Antibacterial evaluation against *Bacillus subtilis*, *Staphylococcus aureus*, and *Escherichia coli* demonstrated heightened sensitivity of bacteria to AgNPs compared to AgNO_3_. Standard antibiotics, tetracycline, and ciprofloxacin, acting as positive controls, exhibited substantial antibacterial efficacy. The green-synthesized AgNPs displayed potent antibacterial activity, suggesting their potential as a viable alternative to conventional antibiotics for combating pathogenic bacterial infections. Furthermore, potential biomedical applications of AgNPs were thoroughly discussed.

## Introduction

The fabrication of nanoparticles derived from noble metals has garnered significant attention in recent decades, with gold and silver emerging as primary candidates for synthesis. Among these, silver nanoparticles (AgNPs) have garnered particular interest due to their exceptional attributes including conductivity, catalytic activity, stability, and antimicrobial properties^1,2^. Notably, AgNPs serve as effective antibacterial, antiviral, and antifungal agents, mitigating surgical infections. Moreover, in contemporary research, AgNPs have emerged as promising candidates for anticancer therapeutics^3,4^, facilitating both diagnosis and treatment across various anticancer potential and apoptosis studies against Pa-1 (Human ovarian teratocarcinoma) cell line^5^.

The use of AgNPs in various fields, particularly in medicine and antimicrobial applications, has gained significant attention due to their unique properties. Pathogenic bacterial infections continue to pose a significant threat to public health, and the growing issue of antibiotic resistance underscores the need for alternative antimicrobial agents. AgNPs have demonstrated noteworthy antibacterial properties, and their green synthesis using plant extracts aligns with the global push for sustainable and eco-friendly practices in nanoparticle production. Nanoparticles possess unique properties and a lot of applications^6–8^. Metal nanoparticles were first investigated in 1887 by Faraday. In the mediaeval era applications of metal nanoparticle were used to decorate the cathedral windows^9^. Due to the unique properties (chemical, electronic, photo-chemical and optical) the search for the novel metal nanoparticles (NMNPs) paced up among the researchers^10–14^. One such nanoparticle is AgNPs that is being used in the clinical care and consumer products due to its toxic properties against pathogens^15,16^. There are many ways of synthesizing nanoparticles^17^. However, the green synthesis of nanoparticles is being used to make variety of nanoparticles due to its more reliable, sustainable and eco-friendly property^18^. Green synthesis involves the use of plant and microorganisms for the synthesis of nanoparticles^19,20^. Nanoparticles biosynthesized from plants is relatively simple and easy process^21^. There are many parameters that affect the synthesis process viz. solvent, temperature, pH and the biological precursor^22^. Plant-derived phytochemicals, such as polysaccharides, alkaloids, terpenoids, flavonoids, saponins, phenolics, and tannins, contribute to the reduction process of nanoparticles. These phytochemicals helps in reducing the metal salts into metal nanoparticles^20,22^. This utilization of environmentally friendly substances reduces reliance on hazardous chemicals, rendering the method reliable, convenient, eco-friendly, and cost-effective^23^. Biologically synthesized plant-derived nanostructures, including crystalline and planar gold nanostructures, spherical AgNPs, spherical indium oxide nanoparticles, and cuboidal palladium nanoparticles, exhibit non-toxicity and stability, offering versatility for applications such as water treatment, drug delivery, biosensing, photocatalysis^24^ and green synthesis and characterizations of AgNPs from *G. glauca* leaf extract^25^ and polysaccharide-capped silver nanoparticles from *Acalypha indica* L.^26^ and evaluation of their bioactive potential.

A spiny herb *Cassia occidentalis* (Family-Caesalpinaceae) was used in the study for green synthesis of AgNPs. The choice of *Cassia occidentalis* L. is rooted in its rich phytochemical composition, particularly polyphenols, which can act as reducing and stabilizing agents in AgNPs synthesis. This plant species produces huge number of seeds (up to 50 seeds per pod) and grow well in shades as well as under open conditions^27^. The seeds are initially soft and juicy and becomes hard after ripening. The plant is found at an altitude of 1000 m in Himalayas, and wild throughout the plains on wastelands or in the coastal area (deltaic region of Western, Eastern and Southern India). The plant has many medicinal properties viz. antidote of poisons, blood purifier, expectorant, anti-inflammatory etc. The formulations such as Liv.52, Himoliv, polyherbal ayurvedic formulations and syrups for liver diseases also contains this the plant extract^28,29^. The plant has its wide use in the fever, gastric discomfort, anemia and general weakness etc.^30,31^. The different plant parts of this species such as flower extract (toxic to *Klebsiella pneumonia*)^32^, leaves (antimalarial, analgesic and antipyretic activity)^33,34^ seeds (antibacterial)^35,36^ were found to have different medicinal effects. The rationale behind this work lies in exploring the antimicrobial potential of AgNPs synthesized from *Cassia occidentalis* L. seeds, with a focus on their efficacy against key bacterial strains. The novelty of this work lies in utilizing *Cassia occidentalis* L. seeds for green synthesis of AgNPs and comprehensively characterizing their structural properties. The study highlights the potent antibacterial efficacy of the green-synthesized AgNPs, positioning them as a promising alternative to conventional antibiotics in addressing pathogenic bacterial infections. The primary objectives of this study are to establish a sustainable method for synthesizing AgNPs through green synthesis using *Cassia occidentalis* L. seed extract. Comprehensive characterization of the AgNPs is conducted using various analytical techniques and AgNPs exhibit potent antibacterial activity against common pathogens, suggesting their potential as an alternative to conventional antibiotics. Additionally, their antioxidant properties further enhance their therapeutic potential, making them promising candidates for biomedical applications.

## Materials and methods

### Plant materials

All materials were sourced from Central Drug House (CDH), including freeze-dried bacterial cultures (*Bacillus subtilis* MTTC-441, *Staphylococcus aureus* MTTC-96, and *Escherichia coli* MTTC-443) obtained from the Institute's microbiology lab. Additionally, *C. occidentalis* seeds were collected from the Moradabad-Delhi highway (Uttar Pradesh, India).

### Extraction of *C. occidentalis* seed aqueous extract

The voucher specimens GEU/sample/019 was deposited and identified by Dr. Sumer Chand, Ex- Scientist C, Systematic Botany Division, Forest Research Institute, Dehradun, India. No, additional approvals were required to conduct research with plant material, in accordance with institutional/local regulations. The powdered material was obtained after the seeds were dried for one week in the dark without humidity. 200 g powder was combined with 2000 mL distilled water (1:10 w/v), heated to 40 °C, agitated for 2 h and kept at ambient temperature for 24 h. The condensed extract was obtained by feeding the main extract into a vacuum distillation equipment (a rotating machine with a vacuum pump) and evaporating the solvent at 40 °C for 1 h. To make extract powder, the condensed solution was heated at 40 °C for 120 h and the resulting product was lyophilized.

### Preparation of AgNPs from *C. occidentalis*

At room temperature, 5 mL extract was added to 45 mL of 0.1 M aqueous AgNO_3_ and agitated for 30 min in the dark. The colour of the solution changed from light yellow to a yellowish brown colour, suggesting the synthesis of AgNPs. The resulting mixture was centrifuged for 20 min at 12,000 rpm to remove the top phase. Washing the silt three times with deionized water and once with ethanol was used to remove any unwanted biological materials.

### Characterization of synthesized AgNPs

#### UV–visible spectroscopy

The biologically synthesized AgNPs from *Cassia occidentalis* L. seeds extract were analyzed using a UV/Visible Spectrophotometer (Shimadzu 1800) to determine their absorption maxima within the range of 300–600 nm. The resulting data were plotted as wavelength (X-axis) against absorbance (Y-axis) on a graph.

#### Scanning electron microscopic analysis

The shape, morphology, and distribution of the synthesized AgNPs were assessed using a SEM. For SEM analysis, a minute amount of AgNPs was placed on conductive carbon tape affixed to an aluminum stub, followed by gold sputtering for 3–4 min.

#### Transmission electron microscopic analysis

The size and surface morphology of the synthesized AgNPs derived from *Cassia occidentalis* L. seeds extract were characterized using a transmission electron microscope. A droplet of the AgNPs solution was deposited onto a carbon copper grid, and images were captured at magnifications ranging from 6000 to 8000× using a Hitachi instrument (Model: S-3400N) operated at 80 kV voltage.

#### Energy dispersive spectroscopy analysis

This method is employed for assessing the elemental composition of substances, such as silver nanoparticles. The sample is inserted into a scanning electron microscope fitted with an EDX. Through EDX analysis, researchers gain crucial insights into the elemental makeup of AgNPs, facilitating the characterization and comprehension of their properties across diverse applications.

#### X-ray diffractometric analysis

The crystalline structure, lattice parameters, and grain size of the synthesized AgNPs were assessed using XRD. The powdered sample of AgNPs was carefully placed in a cavity slide and gently compressed to create a uniform surface. The XRD instrument, operated with data scan software, employed a scan rate of 1.2° per minute. Spectra were recorded within the 5° to 80° range using a CuKα filter (λ = 0.15418 nm) in 2θ/θ scanning mode. The size of the nanoparticles was determined utilizing Scherrer’s formula.

### Evaluation of antioxidant effect of AgNPs from *C. occidentalis*

To assess the antioxidant effects, 2 mL of 100 μM DPPH dissolved in methanol was mixed with 2 mL of different concentrations of AgNO_3_, *C. occidentalis*, and AgNPs. These mixtures were allowed to stand at room temperature for 30 min. Butylated hydroxytoluene (BHT) served as the positive control. Afterward, the absorbance of the samples was measured at 520 nm using a spectrophotometer. The DPPH free radical scavenging percentage was calculated using the following formula:

$${\text{DPPH free radical scavenging }}(\% ) = ({\text{control}} - {\text{test}}) \times 100.$$

Here, the test/control sample comprised 2 mL of DPPH and 2 mL of AgNO_3_, *C. occidentalis*, AgNPs, and BHT at various concentrations, while the control consisted of 2 mL of methanol.

### Analysis of antibacterial properties

For the extract and nanoparticle sensitivity tests,* E. coli, B. subtilis, and S. aureus* were employed. The antibacterial properties were studied using agar disc/well diffusion techniques. A Pasteur pipette was used to form 6 mm wells on the culture medium with consistent spacing in the well diffusion method. In the disc diffusion technique, 6 mm blank discs were used on agar culture medium. The wells and discs were then filled with 60 μL of different dilutions of AgNO_3_, *C. occidentalis* extract and AgNPs. Tetracycline (10 mg/mL) and ciprofloxacin (10 mg/mL) were employed as positive controls in this investigation, with distilled water performing as a negative control (PC-1 and PC-2). After 24 h of incubation at 37 °C, the growth inhibition zone was measured.

### Statistical analysis

The experiments were replicated three times, and the data obtained were entered into STATASTICA 7.0 (STASOFT) for analysis.

## Results

### Synthesis of AgNPs from *C. occidentalis*

During the synthesis of silver nanoparticles, the addition of AgNO_3_ to the prepared extract induced a noticeable shift in the solution’s color, turning it into a yellowish brown color, which indicated the formation of AgNPs. The pH of the reaction mixture was recorded as 8.0. This change in color serves as a primary indicator of nanoparticle formation in the suspension. Following this, the resulting mixture underwent centrifugation at 12,000 rpm for 20 min to facilitate phase separation. The sediment obtained was then washed three times with deionized water and once with ethanol to remove any residual biological impurities effectively.

### UV–visible spectrophotometer

**Figure 1:**
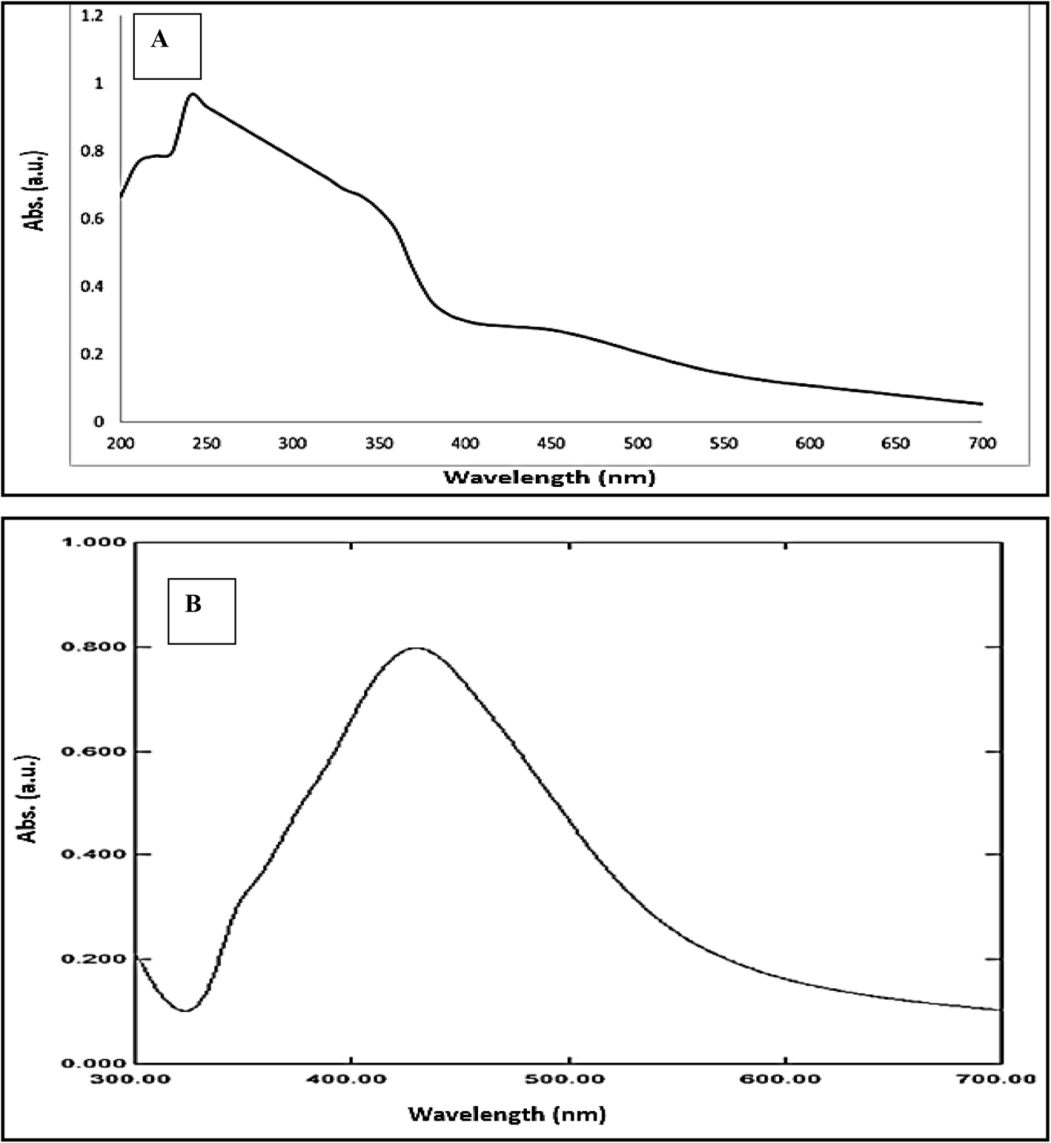
UV–Vis absorption spectra of synthesized (**A**) *C. occidentalis* seed extract (**B**) aqueous solution containing AgNPs.

The AgNPs from *C. occidentalis* were synthesized in solution and confirmed in the 200–700 nm range using a UV–visible spectrophotometer (Shimadzu UV-1800). The spectra of *C. occidentalis* seed extract are shown in Fig. 1A, whereas the spectra of an aqueous solution containing AgNPs are shown in Fig. 1B. The color of AgNPs in aqueous solution was yellowish brown due to that also depends upon the size of particles. There was a single strong peak at 425 nm in AgNPs, but two peaks at 220 and 248 nm in the extract spectra, suggesting he presence of polyphenols and phytochemicals in the solution.

### SEM analysis of AgNPs

**Figure 2:**
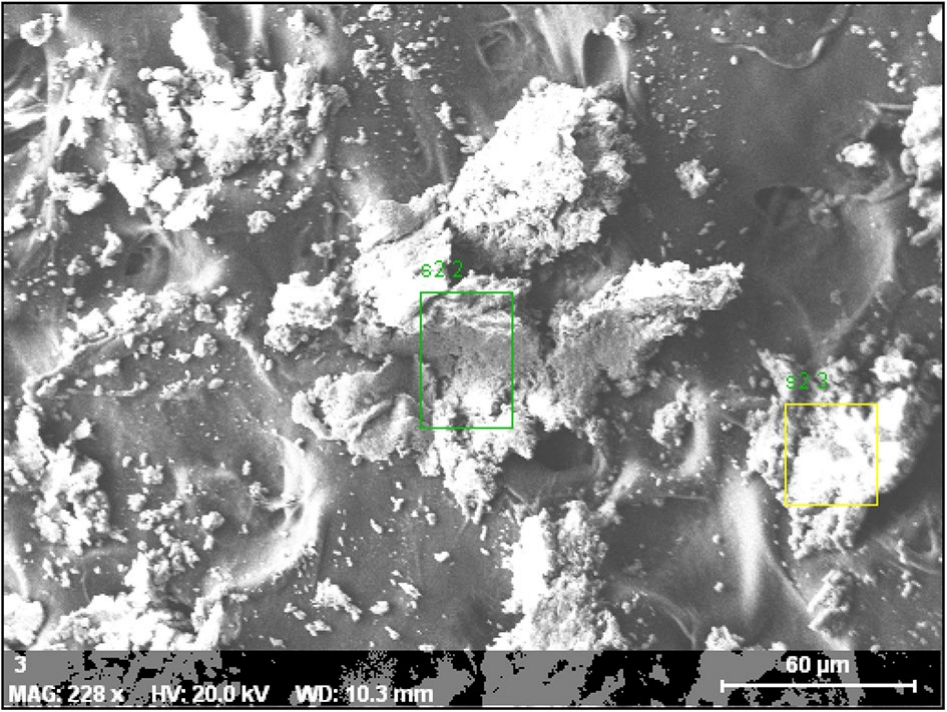
SEM micrograph of synthesized AgNPs.

Surface morphological and nanostructural analyses were conducted using SEM, as depicted in Fig. 2. The SEM micrographs revealed the presence of numerous small aggregates of AgNPs, exhibiting a spherical shape and a polydisperse nature, thus providing insights into the morphology of the AgNPs.

### TEM analysis of AgNPs

**Figure 3:**
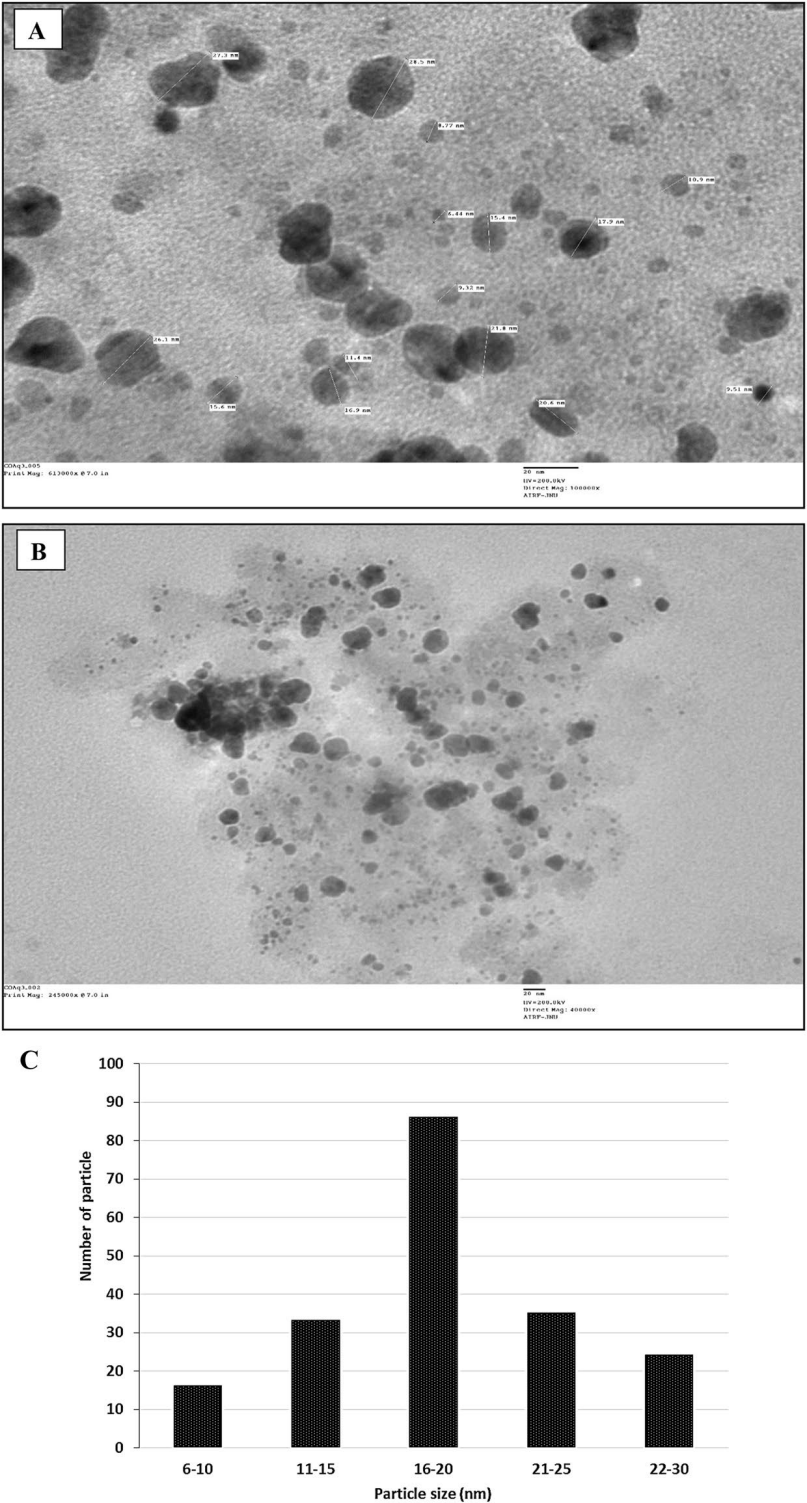
TEM micrograph of the AgNPs using *C. occidentalis* at the scale bar corresponds to (**A**) 20 nm at 100,000× and (**B**) 20 nm at 40,000×. (**C**) Particle size histogram (nm) of AgNPs.

The size and morphology of the nanoparticles were determined using TEM. TEM images depicted the AgNPs as round, spherical, and occasionally oval-shaped, with slight agglomeration observed at specific locations, as illustrated in Fig. 3A,B. Synthesized AgNPs have particle diameters ranging from 6.44 to 28.50 nm. The AgNPs histograms in *C. occidentalis* are shown in Fig. 3C. The size of the particles differs significantly.

### EDX analysis

**Figure 4:**
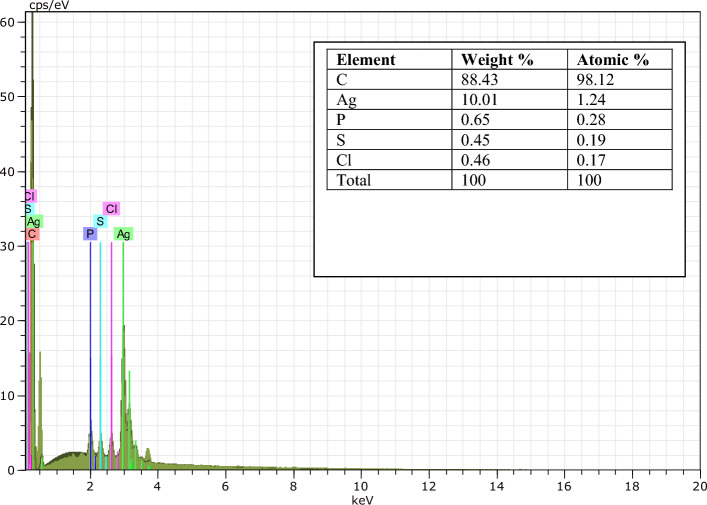
EDX spectrum of synthesized AgNPs.

The elemental composition of AgNPs is presented in Fig. 4, with EDX measurements conducted at 1–10 keV revealing the presence of Ag (10.01%), P (0.65%), S (0.45%), Cl (0.46%) and C (88.43%). The elemental peaks of Ag were identified at both 1 and 3 keV, providing comprehensive insights into the composition of the studied nanoparticles.

### XRD analysis

**Figure 5:**
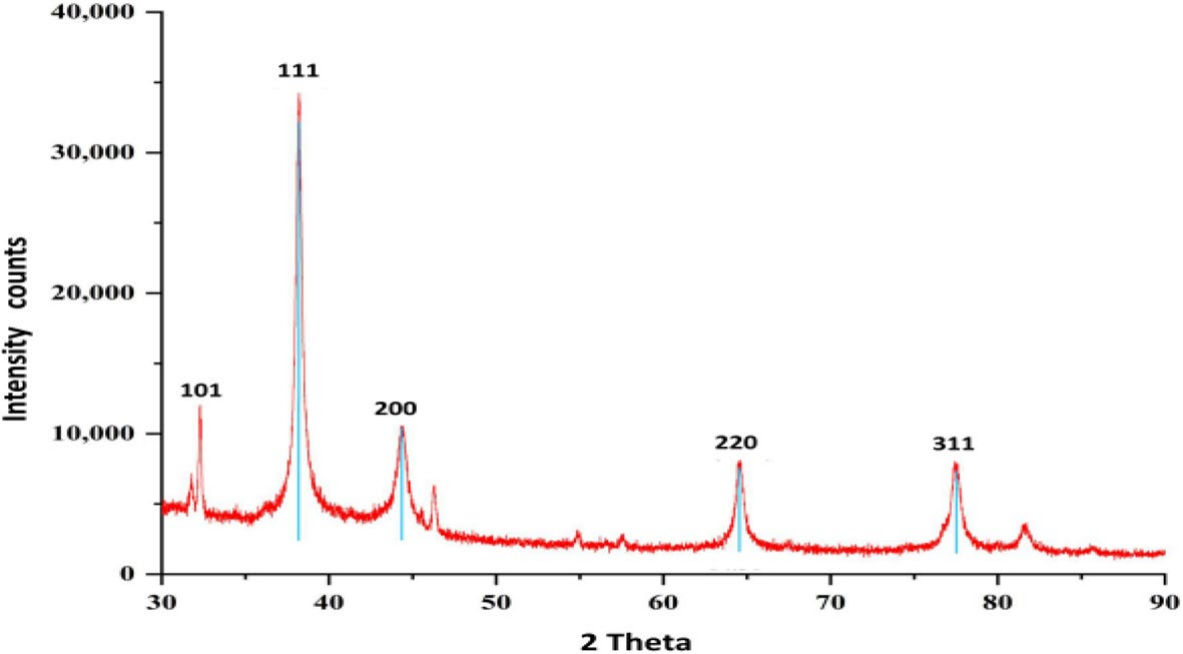
XRD pattern exhibited well-defined Bragg’s reflections at specific 2θ values corresponding to the crystallographic planes 101, 111, 200, 220, and 311, measured at angles of 33.15°, 39.02°, 45.65°, 65.19°, and 78.90°, respectively.

The XRD analysis was employed to investigate the crystalline nature and composition of AgNPs, as well as the phase purity of the synthesized AgNPs. As illustrated in Fig. 5, the XRD pattern exhibited well-defined Bragg’s reflections at specific 2θ values corresponding to the crystallographic planes 101, 111, 200, 220, and 311, measured at angles of 33.15°, 39.02°, 45.65°, 65.19°, and 78.90°, respectively. Additionally, the average particle size of the AgNPs was determined to be 19.78 nm, calculated utilizing Scherr's equation. These results unequivocally confirm the crystalline nature of the AgNPs suspension, thereby providing valuable insights into their structural characteristics and purity.

### Antioxidant activity

**Figure 6:**
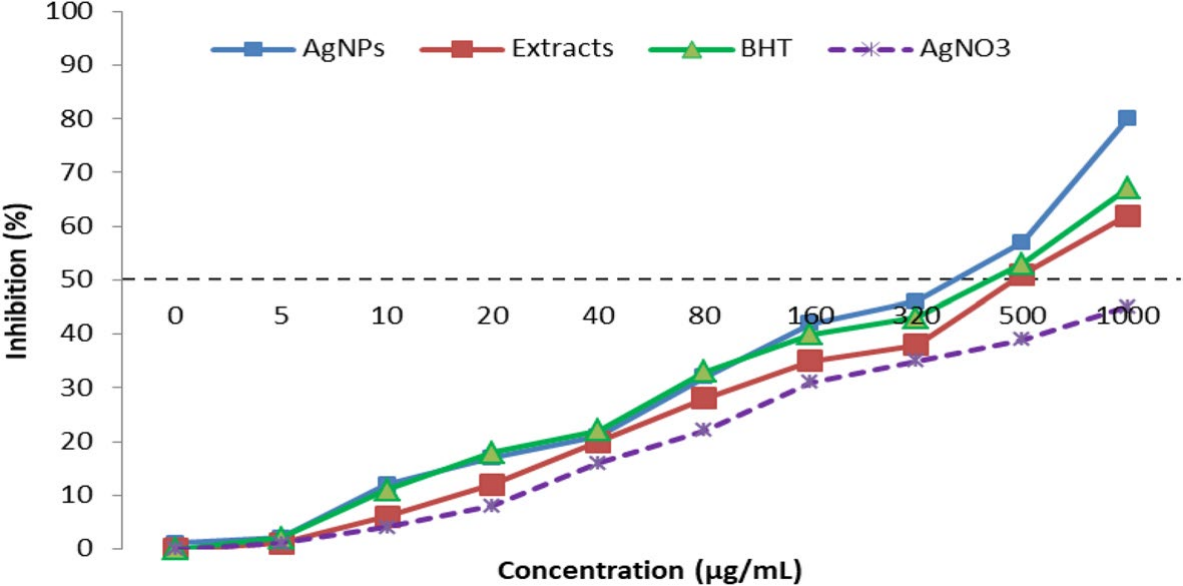
Antioxidant potential of AgNPs, extract of *C. occidentalis,* BHT and AgNO_3_.

The DPPH free radical scavenging activity of AgNPs, *C. occidentalis* seed extract, BHT, and AgNO_3_ was evaluated at various concentrations (0, 5, 10, 15, 30, 60, 120, 240, 500, and 1000 μg/mL). AgNPs exhibited excellent scavenging activity compared to *C. occidentalis* seed extract and AgNO_3_, and was similar to BHT. Specifically, AgNPs, *C. occidentalis* seed extract, and BHT displayed IC_50_ values of 345, 500, and 434 μg/mL, respectively (Fig. 6).

### Antibacterial potential of AgNPs

**Figure 7:**
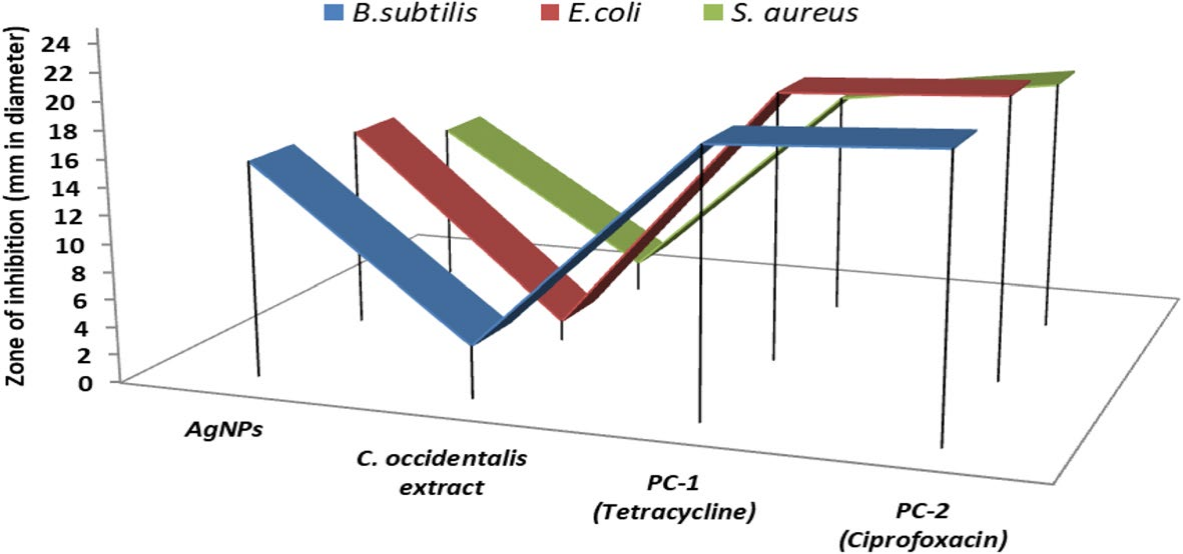
Antibacterial potential of AgNPs and aqueous extracts of *C. occidentalis,* positive controlantibiotic tetracycline and ciprofloxacin against *B. subtilis*, *E. coli* and *S. aureus.*

The antibacterial efficacy of plant-derived AgNPs has been thoroughly explored against various microorganisms, as documented in previous studies^37,38^. In this investigation, three pathogenic bacteria were employed to evaluate the antibacterial properties of AgNPs, plant extract, and standard antibiotics, namely tetracycline and ciprofloxacin (PC-1 and PC-2), as summarized in Table 1. The antibacterial potential of AgNPs and *C. occidentalis* extracts was determined by measuring the diameter of inhibitory zones (mm) against *B. subtilis* (15.50 ± 2.55 and 3.76 ± 2.10), *E. coli* (14.61 ± 0.54 and 01.40 ± 2.90), and *S. aureus* (11.85 ± 1.88 and 02.10 ± 3.50), respectively. For comparison, positive controls consisting of tetracycline and ciprofloxacin standard antibiotics at 15 g/disk demonstrated inhibitory effects against *B. subtilis* (18.90 ± 1.67 and 19.70 ± 1.95), *E. coli* (19.58 ± 0.75 and 20.32 ± 0.42), and *S. aureus* (16.45 ± 1.05 and 18.45 ± 1.23) pathogens, respectively (refer to Fig. 7).

**Table 1:** Antibacterial potential of AgNPs and aqueous extracts of *C. occidentalis* against *B. subtilis*, *E. coli* and *S. aureus.*

S. no.	Name of the organism	Zone of inhibition (mm in diameter)			
		AgNPs	*C. occidentalis* (extracts)	PC-1	PC-2
1	*B. subtilis*	15.50 ± 2.55	3.76 ± 2.10	18.90 ± 1.67	19.70 ± 1.95
2	*E. coli*	14.61 ± 0.54	01.40 ± 2.90	19.58 ± 0.75	20.32 ± 0.42
3	*S. aureus*	11.85 ± 1.88	02.10 ± 3.50	16.45 ± 1.05	18.45 ± 1.23

AgNPs—*Cassia* seed extract-derived silver nanoparticles, *Cassia* seed aqueous extract, PC-(1)—Positive Control (Tetracycline), PC-(2)—Positive control (Ciprofloxacin).

### Possible mechanism of the antibacterial activity of silver nanoparticles

**Figure 8:**
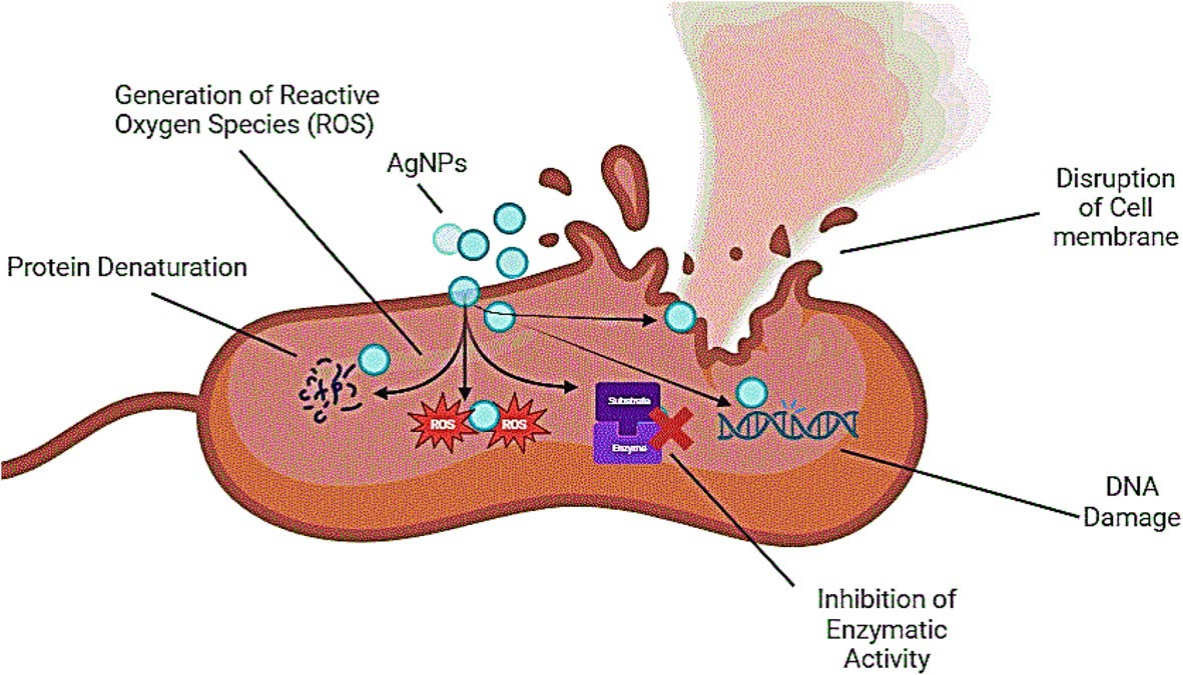
A hypothetical illustration of the possible mechanisms of antibacterial activities of silver nanoparticles against bacterial cells.

Antibacterial properties of AgNPs arise from various mechanisms, as illustrated in Fig. 8.

**A. Disruption of cell membrane:** AgNPs can interact with and disrupt the cell membrane of bacteria. This interaction destabilizes the membrane integrity, leading to leakage of cellular contents and eventual cell death.

**B. Generation of reactive oxygen species (ROS):** AgNPs can induce the generation of reactive oxygen species (ROS) within bacterial cells. ROS, such as superoxide radicals and hydrogen peroxide, cause oxidative damage to proteins, lipids, and DNA, ultimately leading to bacterial cell death.

**C. DNA damage:** AgNPs can penetrate bacterial cells and interact with DNA, leading to DNA damage. This interference with DNA replication and transcription processes can inhibit bacterial growth and viability.

**D. Protein denaturation:** AgNPs can interact with proteins in bacterial cells, leading to their denaturation and loss of function. This disruption of essential cellular processes can impair bacterial growth and survival.

**E. Inhibition of enzymatic activity:** AgNPs can inhibit the activity of essential bacterial enzymes, such as those involved in energy metabolism and cell wall synthesis. This disruption of enzymatic activity can compromise bacterial viability and survival.

## Discussion

The present study describes the biofabrication of AgNPs derived from *Cassia occidentalis* L. seed extract, along with an assessment of their antibacterial, antioxidant properties and potential biomedical applications in the field of anti-diabetic, antiviral, antifungal, antibacterial, DNA cleavage, anti-aging, dye degradation, environmental assay indicators, plant growth, and antioxidants. In recent advancements in nanoscience, various approaches have been employed to synthesize AgNPs from different plant components. Initially, the synthesis of AgNPs from *C. occidentalis* L. seed extract was indicated by a noticeable change in color when the seed extract was combined with AgNO_3_ at a ratio of 1:9 (v/v). The shift from light yellow to dark brown served as an indicator of the surface plasmon resonance (SPR) of metallic silver, indicating the formation of AgNPs. This synthesis process suggested that the plant extract, rich in diverse phytoconstituents, acts as both reducing and capping agents^39^. The pH of the reaction mixture was recorded as 8.0 at that point, aligning with findings from other studies. The highest SPR absorption, observed at 425 nm, indicated that the reaction was concluded when the color transitioned from light yellow to dark yellowish brown. The excitation of the UV–visible band imparts a yellowish-brown color to AgNPs in aqueous solution, with the specific shade dependent on the particle size^29^. In previous studies, the SPR range of silver nanoparticles, typically between 410 and 450 nm, was associated with spherical nanoparticles^40,41^, As per publications, another study^42^ identified a peak at 461.02 nm during the synthesis of AgNPs using the seed extract of *C. occidentalis.* Similarly, the utilization of *Pyrostegia venusta* and *Passiflora vitifolia* leaf extracts, containing a variety of phytochemicals, has been proposed for AgNPs synthesis^43,44^.

The SEM micrographs revealed the presence of numerous small aggregates of AgNPs, exhibiting a spherical shape and a polydisperse nature, thus providing insights into the morphology of the AgNPs. Anandalakshmi et al. reported similar shapes, observing even-shaped, spherical AgNPs in SEM images derived from biosynthesized AgNPs from *Pedalium murex* leaf extract^45^. Hemalata et al. also noted comparable shapes in SEM images of biosynthesized AgNPs from a *Cucumis prophetarum* leaf extract^46^.

The TEM images depicted the AgNPs as round, spherical, and occasionally oval-shaped, with minor agglomeration observed at specific sites. Particle sizes ranged from 6.44 to 28.50 nm. As per other reports, AgNPs synthesized from *D. indica* exhibited a spherical morphology with size ranges of 10.0 to 23.24 nm. Although aggregation was evident in the AgNPs, a small fraction displayed dispersion and variations in size^39^. Previous studies indicated that AgNPs derived from *I. balsamina* and *L. camara* leaf extracts exhibited spherical shapes with size ranges of 10–30 nm and a polydisperse nature^47^.

The EDX spectra of AgNPs indicated that the sample comprised 10.01% of silver, with a significant peak observed at 3 keV, suggesting the reduction of Ag^+^ ions to Ag°. Additionally, the EDX spectrum revealed the presence of carbon, sodium, chlorine, and other elements, along with the identification of supplementary metallic elements. Similarly, AgNPs derived from *R. serrata* flower buds extract exhibited a prominent signal for silver, as well as elemental peaks corresponding to phytomolecules, with additional peaks of carbon and oxygen observed^48^. The EDX analysis of AgNPs demonstrated a notable signal for silver, along with other elemental peaks. These additional peaks, apart from silver, may be attributed to the presence of phytomolecules on the external surface of the nanoparticles, playing a crucial role in capping and stabilization. Peaks indicating carbon, oxygen, and other elements may be attributed to atmospheric moisture content.

X-ray diffractometry confirmed the face-centered cubic crystal structure of AgNPs. The XRD patterns exhibited reflection peaks at 33.15°, 39.02°, 45.65°, 65.19°, and 78.90° 2 theta, corresponding to the 101, 111, 200, 220, and 311 Bragg’s plane faces, respectively. These results indicate the crystalline nature of the AgNPs suspension, consistent with findings from other studies. Another investigation into the production of silver nanoparticles from *C. sativus* revealed a similar XRD pattern, with crystalline phases associated with inorganic plant extract components present on the surface of the synthesized AgNPs^5^.

The antioxidant potential of the synthesized AgNPs, aqueous *C. occidentalis* seed extract, butylated hydroxytoluene (BHT), and AgNO_3_ was investigated using the DPPH free radical assay, a widely recognized method for assessing antioxidant activity. DPPH, being a stable compound, serves as a valuable tool in evaluating antioxidant capacity, as it readily accepts hydrogen or electrons. The IC_50_ value obtained from this assay serves as an indicator, with lower values indicating stronger DPPH scavenging activity. Our findings revealed that both the synthesized AgNPs and the aqueous extract possess significant free radical scavenging abilities. Interestingly, AgNPs exhibited remarkable scavenging activity, comparable to that of BHT, and surpassed *C. occidentalis* seed extract and AgNO_3_. These findings are consistent with previous research demonstrating the considerable antioxidant properties of Ag nanoparticles, which effectively neutralize various free radicals, including DPPH^49,50^. Antioxidants play a crucial role in combating free radicals^51^. The DPPH antioxidant assay is a well-established method known for its ability to assess the capacity of compounds to reduce free radicals^52,53^. Stable free radical scavengers, such as DPPH, exhibit an absorbance at 517 nm and undergo a color change from violet to yellow during the reduction process^54^. Free radicals induce cellular damage, posing health risks to both humans and animals^55^.

According to the findings, AgNPs are a good material for use as antibacterial agents against pathogenic bacterial species as also evident by previous research^56–58^. Recent findings indicate that silver and copper nanoparticles possess biocidal properties, making them suitable for use as antibacterial coatings on consumer goods^59^. Research has demonstrated that silver nanoparticles can serve as effective antibacterial agents against both gram-positive and gram-negative bacterial infections^60,61^. Silver nanoparticles interact with the bacterial cell wall in a natural manner, disrupting its integrity and causing the breakdown of phosphodiester linkages, ultimately leading to the bacterium's demise. Additionally, silver ions bind to crucial biological components such as sulfur, oxygen, and nitrogen, thereby impeding bacterial growth^62^.

### Potential biomedical applications

The versatility of AgNPs in diverse applications, including anti-diabetic, antiviral, antifungal, antibacterial, DNA cleavage, anti-aging, dye degradation, environmental assay indicators, plant growth, and antioxidants, as well as their protective role, is illustrated in Table 2. For example, AgNPs have exhibited promise in regulating glucose levels, presenting therapeutic advantages in diabetes management. Research suggests that AgNPs can modulate insulin signaling pathways, potentially enhancing insulin sensitivity and cellular glucose uptake, thus offering a novel approach for treating diabetes^63,64^. AgNPs also demonstrate significant antiviral properties by disrupting viral attachment and entry into host cells. This mechanism has been investigated against viruses like HIV, influenza, and herpes simplex virus, indicating potential applications in the development of antiviral agents and coatings for medical equipment to mitigate viral transmission^65,66^. The antifungal efficacy of AgNPs has been proven against various fungal pathogens, including *Candida* species. This suggests their potential utility in antifungal formulations for the treatment of fungal infections, especially in topical applications^67–69^. Renowned for their potent antibacterial properties, AgNPs demonstrate effectiveness against both Gram-positive and Gram-negative bacteria. This renders them promising candidates for the development of antimicrobial coatings, wound dressings, and antibacterial agents in medical settings^70,71^. The capability of AgNPs to cleave DNA strands holds significant implications for genetic and molecular research, offering potential applications in targeted drug delivery, gene therapy, and as a tool for understanding DNA structure and function^72,73^. The anti-aging properties of AgNPs, attributed to their capacity to scavenge free radicals and alleviate oxidative stress, present opportunities for potential utilization in skincare formulations. This application could aid in diminishing signs of aging, including wrinkles and fine lines^74^. In environmental contexts, AgNPs have shown the capability to degrade synthetic dyes, proving valuable in environmental remediation endeavors. Potential applications include wastewater treatment, where AgNPs could be employed to break down harmful dyes and pollutants^75,76^. Utilized as indicators in environmental assays, AgNPs are employed to evaluate their impact on ecosystems. Studies utilize various biological indicators to comprehend how AgNPs may affect aquatic and terrestrial environments, thereby assisting in the management of potential ecological risks^77^. Research examining the influence of AgNPs on plant growth parameters suggests potential agricultural applications. AgNPs have been investigated for their effects on seed germination, root development, and overall plant growth, hinting at a possible role in enhancing crop yields^78,79^. Moreover, AgNPs may demonstrate antioxidant properties, shielding cells from oxidative stress. This antioxidant capacity could hold implications for health supplements and dietary applications, contributing to overall cellular health^80,81^.

**Table 2:** Impact of silver nanoparticles from *Cassia occidentalis* L. and seed extract on multiple functions, including anti-diabetic, antiviral, antifungal, antibacterial, dna cleavage, anti-aging, dye degradation, environmental assay indicators, plant growth and antioxidants and protective role.

S. no	Functionality	Effect of AgNPs and seed extract of *Cassia occidentalis* L	References
1	Anti-diabetic	Potential impact on glucose regulation and diabetes management	^64,82,83^
2	Anti-viral	Evaluation of antiviral activity against specific viral strains	^65,66^
3	Antifungal	Examination of antifungal properties, targeting fungal pathogens	^67,68,84,85^
4	Antibacterial	Assessment of antibacterial efficacy against diverse bacterial species	^86^
5	DNA cleavage	Investigation of DNA cleavage ability, exploring potential applications in genetic research	^87,88^
6	Dye degradation	Study on the capacity of AgNPs to degrade synthetic dyes, indicating potential for water treatment	^89–91^
7	Antiaging	Exploration of antiaging effects, focusing on cellular and molecular impacts	^73,74,92,93^
8	Environmental assays	Evaluation of indicator species to gauge environmental impact and toxicity	^94,95^
9	Plant growth	Examination of influence on plant growth parameters, including germination and biomass	^79,96^
10	Antioxidants and protective role	Oxidative stress-induced DNA and cell membrane damage	^56,80,97,98^

The novelty of this work lies in the eco-friendly synthesis of AgNPs using *Cassia occidentalis* L. seed extract, offering a sustainable alternative to traditional chemical synthesis methods. Additionally, the comprehensive characterization of the synthesized AgNPs, coupled with the assessment of their antioxidant properties and antibacterial efficacy, highlights their multifunctionality and potential as versatile biomedical agents. This integrated approach expands the scope of research in the field by providing insights into the synthesis, characterization, and potential applications of green-synthesized AgNPs in bacterial treatment and beyond.

## Data Availability

All the data generated/analyzed during the study are available with the corresponding author on reasonable request.
